# Celastrol Induces Apoptosis in Gefitinib-Resistant Non-Small Cell Lung Cancer Cells via Caspases-Dependent Pathways and Hsp90 Client Protein Degradation

**DOI:** 10.3390/molecules19033508

**Published:** 2014-03-21

**Authors:** Xing-Xing Fan, Na Li, Jian-Lin Wu, Yan-Ling Zhou, Jian-Xing He, Liang Liu, Elaine Lai-Han Leung

**Affiliations:** 1 State Key Laboratory of Quality Research in Chinese Medicine, Macau Institute For Applied Research in Medicine and Health, Macau University of Science and Technology, Macau SAR, China; 2 Guangzhou Institute of Respiratory Disease, State Key Laboratory of Respiratory Disease, The 1st Affiliated Hospital of Guangzhou Medical College, Guangzhou 510120, China

**Keywords:** celastrol, gefitinib resistance, NSCLC, apoptosis, Hsp90, EGFR

## Abstract

Celastrol, a triterpene extracted from the Chinese herb *Tripterygium wilfordii*, has been shown to have multiple bioactivities. Although among these activities, its anti-cancer effects have attracted the most attention, the effect of celastrol on gefitinib-resistant non-small cell lung cancer (NSCLC) cells is not clearly known. Here, we examined the potency of celastrol in three different NSCLC cell lines. We explored its treatment mechanism in two gefitinib-resistant NSCLC cell lines (H1650 and H1975). Our data demonstrated that celastrol exerted its apoptotic effect in a dose- and time-dependent manner. Also, the mitochondria membrane potential was gradually lost and the ratio of Bax/Bcl-2 increased after the treatment of celastrol, both of which are indicators of mitochondria membrane integrity. Although the caspases were activated, the treatment with pan-caspase inhibitor could partially inhibit the level of apoptosis. Moreover, the protein level of Hsp90 client proteins, EGFR and AKT, was measured. Interestingly, both client proteins were remarkably down-regulated after the treatment of celastrol. Taken together, our data showed that celastrol may be developed as a promising agent for treating gefitinib-resistant NSCLCs by inducing apoptosis through caspase-dependent pathways and Hsp90 client protein degradation.

## 1. Introduction

Celastrol, a triterpene derived from the Chinese medical herb *Trypterygium wilfordii* [1], has been shown to be an effective treatment in multiple complex diseases, such as rheumatoid arthritis [2], lupus erythematosus [3], lateral sclerosis [4], Alzheimer’s disease [5] and asthma [6]. However, the most attractive function of celastrol is its anti-cancer effect. It has been shown to have very powerful anti-cancer activity in many kinds of cancer cell types with various treatment mechanisms. For example, it inhibited the growth of tumor cells by suppressing angiogenesis in prostate cancer [7] and it inhibited IκBα kinase activation [8], proteasome activity [9], or vascular endothelial growth factor receptor (VEGFR) expression [10] to induce apoptosis. However, whether it exhibits selective anti-cancer activity in gefitinib-resistant NSCLC still remains uncertain.

Lung cancer remains the leading cause of cancer deaths globally [11]. Patients with NSCLC, which accounts for 80% of all lung cancer cases, are often diagnosed at advanced stages of the disease, thus the prognosis of lung cancer remains poor [12]. With the development of advanced DNA sequencing technology, the therapeutic strategy of NSCLC has been modified towards to personalized therapy. Some specific driver genetic mutations have been identified in NSCLC, such as *EGFR* [13,14], *EML4-ALK* fusion gene [15] and *ROS* fusion gene [16], which direct the development of anti-cancer drugs towards to molecular-targeted therapy, by specifically targeting these driver mutations for individualized treatment purpose. EGFR protein overexpression was observed in 62% of the NSCLC patients and correlated with poor prognosis [17,18]. Owing to the strong tumor-promoting effect of *EGFR* mutation and *EGFR* overexpression, specific small molecule inhibitors directly targeting EGFR are being developed. For example, gefitinib which is a tyrosine kinase inhibitor (TKI) can specifically inhibit EGFR and its downstream survival signaling pathway [19]. NSCLC patients with L858R point mutation or exon 19 deletion in *EGFR* have been reported to show good initial responses to gefitinib [20]. However, despite the good initial significant response of TKI, like other chemotherapeutic agents, patients acquire resistance to TKI ultimately. The median time to disease progression is about 12 months. Although the reason of drug resistance is multi-factorial, 49% of the gefitinib-resistant cases are associated with double mutation on *EGFR* [21], the remaining resistant cases are due to the presence of other driver mutation genes which lead to bypass of the gefitinib treatment. Therefore, it is necessary and urgent to discover more effective potential agents as candidate drugs for treating gefitinib resistant NSCLC patients. In this study, we applied three different NSCLC cell lines, which are H1650, H1975 and H2228 for evaluating the effect of celastrol. H1650 and H1975 display primary resistance to gefitinib [22]. H1650 has an activating deletion mutation on exon 19 of the *EGFR* gene [20,23] and other driver mutation genes which leads to gefitinib resistance [24]. H1975 habors T790M mutation in addition to the L858R activating mutation which is directly associated with gefitinib resistance, the steric hindrance provided by the bulky methionine amino acid residue hinders the binding of gefitinib to EGFR [25]. H2228 contains wild-type EGFR and EML4-ALK fusion gene mutation [26] and could be used as EGFR survival independent control cells. We have attempted to examine the levels of the apoptotic effect exerted by celastrol and study the anti-cancer mechanism of celastrol on the two gefitinib-resistant NSCLC cell lines, H1650 and H1975. 

## 2. Results and Discussion

### 2.1. Celastrol is More Effective in Decreasing Cell Viability of Gefitinib-Resistant H1650 and H1975 Cells

**Figure 1:**
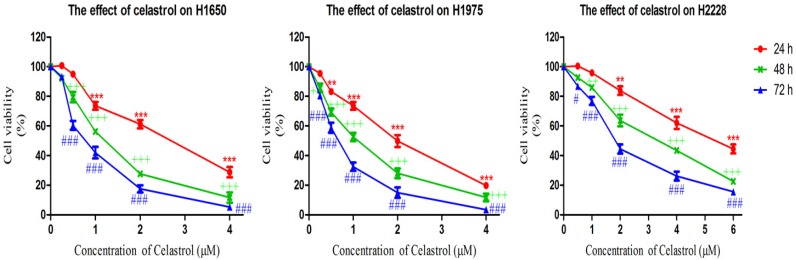
MTT assay results showed that celastrol decreased the cell viability of H1650, H2228 and H1975 after 24, 48, 72 h treatment. Results are expressed as mean ± S. E. (*n* = 3, * *p* < 0.05, ** *p* < 0.01, *** *p* < 0.001 for 24 h analysis; + *p* < 0.05, ++ *p* < 0.01, +++ *p* < 0.001 for 48 h analysis; # *p* < 0.05, ## p < 0.01, ### *p* < 0.001 for 72 h analysis).

The cytotoxicity of celastrol was determined by MTT assay. We examined the effect of celastrol on three NSCLC cell lines, H1650, H2228 and H1975. Both H1650 and H1975 harbor EGFR-activating mutations but are resistant to gefitinib [22], while H2228 harbors a wild-type EGFR that can be used as EGFR independent control cell line [26]. Cells were incubated with a range of celastrol concentrations (0, 0.25, 0.5, 1, 2, 4, 6 μM) for 24, 48, and 72 h and cell viability assays were performed. As shown in Figure 1, although the viability of the three NSCLC cell lines were all significantly inhibited by the treatment of celastrol in a dose- and time-dependent manner, the two gefitinib resistant cell lines were more sensitive to the treatment. The IC_50_ values of celastrol on the three cell lines at 24, 48 and 72 h are shown in Table 1. The results mentioned above suggested that celastrol exhibited stronger cytotoxic effect on gefitinib-resistant NSCLC H1650 and H1975 cells, when compared with H2228 which contains wild-type EGFR. The IC_50_ value of celastrol in H2228 is nearly 3-fold higher than that of H1975 and H1650. 

**Table 1:** The IC_50_ values of celastrol on three NSCLC cell lines.

Cell line	IC_50_ Value (μM)		
	24 h	48 h	72 h
**H1650**	2.78 ± 0.77	1.18 ± 0.32	0.79 ± 0.27
**H1975**	2.03 ± 0.48	1.00 ± 0.18	0.60 ± 0.15
**H2228**	>6	3.28 ± 0.74	1.95 ± 0.45

### 2.2. Celastrol Induced Apoptosis in H1650

**Figure 2:**
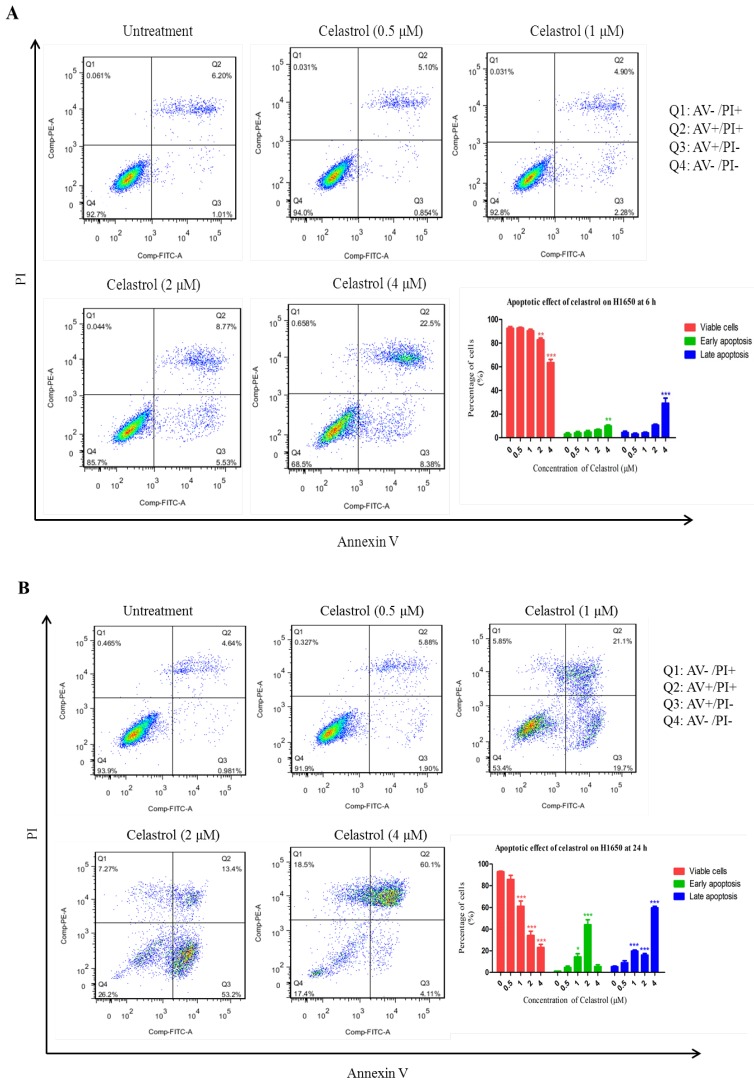
Celastrol induced apoptosis in H1650 cells at different time points. (**A**) After treating with celastrol (0.5, 1, 2, 4 μM) for 6 h, cells were collected for apoptosis analysis. (**B**) Cells were collected for apoptosis analysis at 24 h after the treatment of celastrol (0.5, 1, 2, 4 μM).

To our knowledge, the anti-cancer effect of celastrol on H1650 has not been reported, but this cell line represents a specific group of NSCLC patients with resistance to gefitinib but without a EGFR double mutation. To examine whether celastrol induces cytotoxicity and apoptosis in H1650 cells, cells were treated with the indicated concentrations of celastrol for 6 and 24 h respectively, and stained with Annexin V-FITC/PI, wheras Annexin V-stained cells and double-stained cells are the early and late stage of apoptotic cell respectively. Figure 2A,B show the apoptosis analysis results of the treated-cells and the control cells after treatment for 6 and 24 h, respectively. The flow cytometry results showed that celastrol induced a significant increase in apoptosis as early as 6 h, indicating that celastrol is a fast and strong apoptosis inducer. For example, after 6 h treatment, more than 30% of the cells treated with 4 μM of celastrol underwent apoptosis (Q2 + Q3). When the cells were treated with 4 μM of celastrol for 24 h, the percentage of apoptotic cell sharply increased to 64.2%. These results suggested that celastrol can quickly and significantly induced apoptosis in H1650. 

### 2.3. Celastrol Induced Apoptosis via Mitochondria-Dependent Pathway

**Figure 3:**
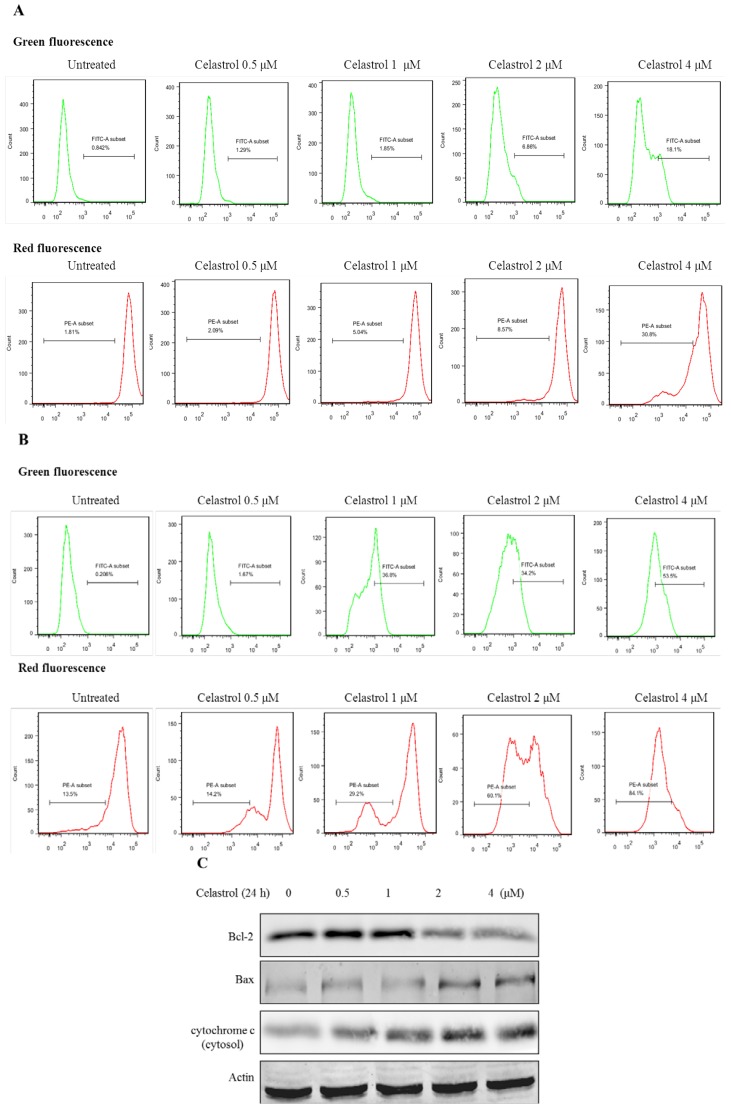
Celastrol induces apoptosis via mitochondria-dependent pathway. (**A**) The level of MMP of H1650 cells was analyzed after the treatment of celastrol for 6 h by flow cytometry. (**B**) The level of MMP of H1650 cells was analyzed after the treatment of celastrol for 24 h by flow cytometry. (**C**) Celastrol regulated the ratio of Bcl-2/Bax and caused cytochrome C release from the mitochondria.

To investigate whether the apoptotic effect induced by celastrol in H1650 was mediated through mitochondria dependent pathways, the mitochondria membrane potential (MMP) was measured by using the fluorescence dye, JC-1. It was known that the loss or disruption of MMP during the early phase of apoptosis results in an increase in mitochondria membrane permeability, and release of cytochrome c into the cytosol, which further leads to activation of caspases, finally causing apoptosis [27]. Changes in MMP of H1650 cells were detected at 6 and 24 h after treatment of celastrol, as shown in Figure 3A,B. Because JC-1 has two different forms, the monomer and the aggregate form. If MMP is gradually lost and depolarized, JC-1 will predominantly exist as the monomer form which yields green fluorescence signals. On the other hand, JC-1 will aggregate together and then yield red fluorescence in normal hyperpolarized mitochondria [28]. Compared with the untreated cells, celastrol showed the capability of inducing a significant loss of MMP, which was indicated by the higher green fluorescence but lower red fluorescence signals. 

Next, we examined whether the loss of MMP would mediate the ratio of Bcl-2/Bax and cytochrome c in celastrol-treated H1650 cells. As shown in Figure 3C, following treatment of celastrol, the protein level of Bcl-2 remarkably decreased, while that of Bax increased. Cytochrome c was gradually released from mitochondria to the cytosol. These results suggested that the apoptosis induced by celastrol was mediated through mitochondria-dependent pathways as well as changes in the expression of Bcl-2 family proteins.

### 2.4. Caspases were Significantly Activated by the Treatment of Celastrol and Inhibition of Caspases could Partially Attenuate the Apoptotic Effect Induced by Celastrol

**Figure 4:**
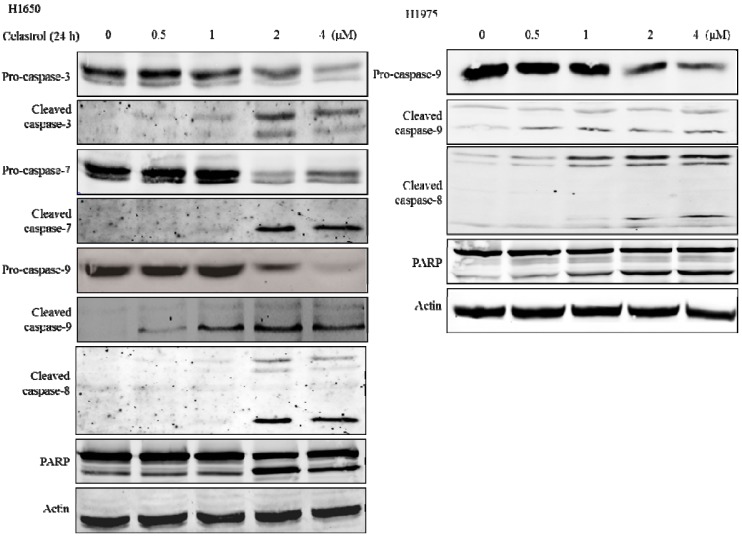
Effect of celastrol on the activation of caspase 3, 7, 8, 9 and PARP was detected at 24 h after the treatment of celastrol, and β- actin was considered as a loading control. Left panel showed the Western blot results of H1650, and the right panel showed the Western blot results of H1975.

The activation of caspases is essential for both the extrinsic and the intrinsic apoptotic pathways [29], and caspase 3/7 are considered as the executors of apoptosis [30]. Once they are activated, this will lead to the execution stage of apoptosis [31]. To understand the role of caspases in the process of celastrol-induced apoptosis, we examined the effect of celastrol on the activation of caspases. In Figure 4, western blot results showed that celastrol was able to effectively induce the cleavage of caspases 3 and 7 at the concentrations of 2 and 4 μM. PARP, which is involved in the repair of DNA damage in response to apoptotic signal stimulation and is the downstream effector of caspases [29], was also significantly activated. These suggest that celastrol significantly stimulated the activation of caspases in H1650. 

Then we tested whether the induction of apoptosis by celastrol is associated with extrinsic or intrinsic apoptosis pathway. We found that both caspase-8 and caspase-9 which are the key components of extrinsic apoptosis pathway [32] and intrinsic apoptosis pathway, respectively [33], were significantly activated by the celastrol treatment, which suggested that the apoptotic effect of celastrol is mediated by both intrinsic and extrinsic apoptosis pathway. Similar results were observed in H1975, which is another gefitinib-resistant NSCLC cell line. As shown in the right panel of Figure 4, both caspase-8 and 9 were activated by the treatment of celastrol. Moreover, PARP, a marker of apoptosis was also significantly activated. Therefore, celastrol can induce both extrinsic and intrinsic apoptosis pathways in gefitinib-resistant NSCLC cells.

**Figure 5:**
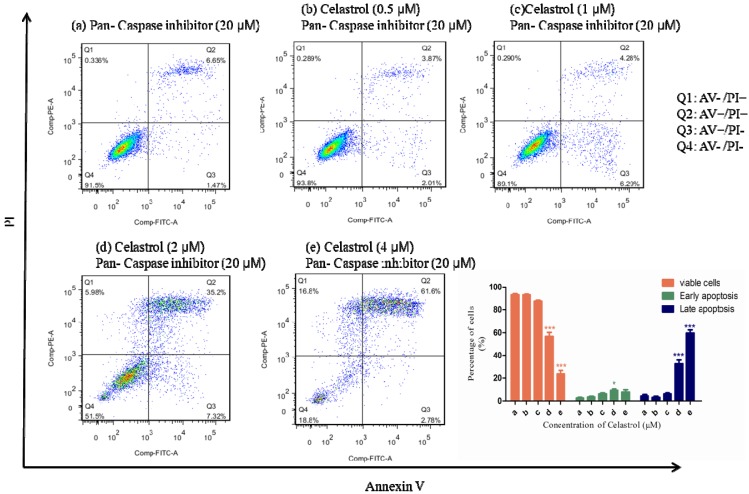
Compared with the celastrol single treatment group, the pretreatment of pan-caspase inhibitor can partially attenuate the apoptotic effect induced by celastrol in H1650.

To further explore whether caspases activation is necessary for the induction of apoptosis, we pretreated cells with the pan-caspase inhibitor Z-VAD-fmk at 20 μM for 1 h and then co-treated with celastrol for 24 h. Interestingly, except for the high dosage group (4 μM), the pretreatment of pan-caspase inhibitor can partially inhibit the apoptosis induced by celastrol. By comparing the results of Figure 2B and Figure 5, after the pre-treatment of pan-caspase inhibitor in the 1 and 2 μM celastrol treatment groups, it showed that the percentage of apoptotic cells significantly decreased. However, in 4 μM celastrol treatment group, there was no significant change. It indicated that the effect of apoptosis-inhibition effect of pan-caspase inhibitor cannot totally offset the effect of apoptosis induced by of celastrol in H1650. Taken together, these results indicated that caspases were significantly activated by the treatment of celastrol and the inhibition of caspases activation can partially attenuate the celastrol-induced apoptosis in H1650.

### 2.5. Celastrol Down-Regulated Hsp90 Client Proteins

Since inhibition of caspases was unable to fully block the apoptotic effect of celastrol, there should be alternative pathway that can mediated celastrol-induced apoptosis. Celastrol has been identified as a Hsp90 inhibitor and apoptosis mediator by facilitating the degradation of Hsp90 client proteins [34]. It also stabilizes a number of proteins required for tumor growth, therefore Hsp90 inhibitors are highly investigated as anti-cancer drugs, especially for drug-resistant cancer [35]. 

**Figure 6:**
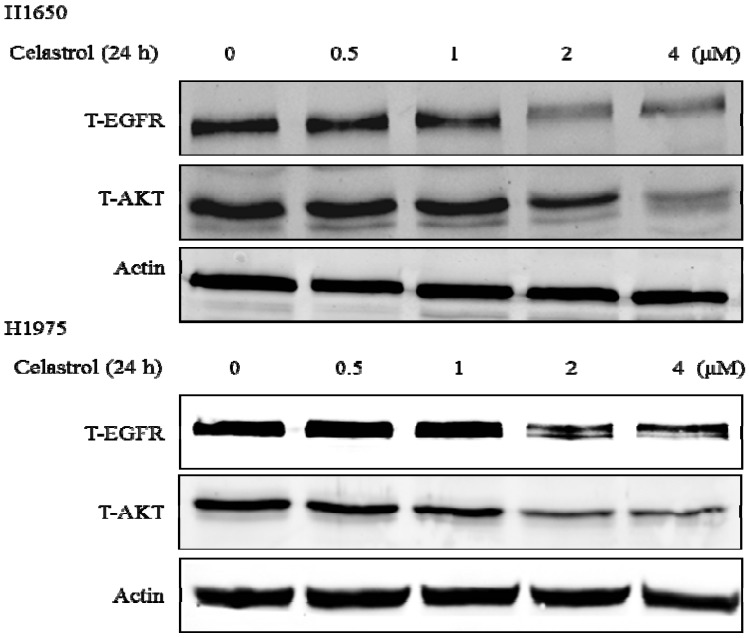
Celastrol significantly induced the degradation of Hsp90 client proteins: EGFR and AKT in both H1650 and H1975.

To determine whether celastrol can regulate these chaperone proteins in H1650 and H1975, we measured the protein level of EGFR and AKT, two well-known client proteins of Hsp90. As shown in Figure 6, both proteins were significantly down-regulated by the treatment of celastrol starting at 2 μM. These results indicated that celastrol can inhibit Hsp90 chaperone function in these two gefitinib-resistant cell lines. Since the EGFR activated mutation was considered to be one of the main reasons of the carcinogenesis of H1650 and H1975 [36], the inhibition of Hsp90 client proteins induced by celastrol plays a tumor suppressing role in this EGFR survival dependent cells. 

## 3. Experimental

### 3.1. Materials

Celastrol powder was purchased from Sigma Aldrich (St. Louis, MI, USA). The primary anti-bodies of anti-actin, total EGFR, total/phospho- AKT, PARP, caspase-3, caspase-7, cleaved caspase-8 and caspase-9 were purchased from Cell Signaling Technology (Danvers, MA, USA). The primary anti-bodies of anti-Bcl-2, Bax, cytochrome c were purchased from Santa Cruz (Dallas, Texas, TX, USA). Fluorescein-conjugated goat anti-rabbit and mouse anti-bodies were purchased from Odyssey (Belfast, ME, USA). Pan-caspase inhibitor, JC-1, and Annexin V/ PI staining dye were purchased from BD Biosciences (San Jose, CA, USA). 

### 3.2. Cell Lines and Cell Culture

H1650, H1975 and H2228 were purchased from ATCC and cultivated with RPMI 1640 medium supplemented with 10% fetal bovine serum (Gibco, Big Cabin, Oklahoma, ME, USA), 100 U/mL penicillin and 100 μg/mL streptomycin (Gibco, Big Cabin, Oklahoma, ME, USA). All the cells were cultivated at 37 °C with 5% CO_2 _incubator.

### 3.3. 3-(4, 5-Dimethylthiazol-2-yl)-2, 5-diphenyltetrazolium bromide (MTT) Assay

Cells were seeded on a 96-well microplate with 4000 cells/well, and were cultured overnight for cell adhesion. Wide concentrations of range of celastrol were added with vehicle control: Dimethyl sulfoxide (DMSO) and the microplates were incubated for 24 or 48 h. Each dosage was repeated as triplicate. Ten μL of MTT (5 mg/mL) solution was added to every well and the plate was placed back in the incubator for 4 h. Then 100 μL of the resolved solution (10% SDS and 0.1 mM HCL) was added to each well and the plate was further incubated at 37 °C for another 4 h to dissolve the formazan crystals. Finally, the absorbance of the plate was measured at 570 nm (absorbance) and 650 nm (reference) by a microplate reader (Tecan, Morrisville, NC, USA). The cell viability is calculated as the percentages change of the absorbance of treated cells divided by the absorbance of untreated cells. 

### 3.4. Assessment of Apoptosis Levels by Annexin V/PI Staining

After treatment, cells were harvested, washed with PBS, and re-suspended with 1 x binding buffer. Then cells were double-stained with Annexin V/PI for 15 min at room temperature in dark. Apoptotic cells were quantitatively counted by a flow cytometer (BD FACSAria III). 

### 3.5. Analysis of Mitochondrial Membrane Potential

Cell permeable and mitochondrial specific fluorescent dye JC-1 was applied to determine the mitochondrial membrane potential (MMP) of H1650. After treatment, cells were harvested, washed, and stained with JC-1 for 15 min in the incubator at 37 °C. Then cells were washed again and suspended with regular medium. The change in MMP of the cells was measured by flow cytometer followed with the manufacturer’s protocol. In brief, the fluorescence emission spectrum of JC-1 is dependent on its concentration. There are two forms of JC-1 which are the monomer and the aggregate forms. If MMP is gradually lost and depolarized, JC-1 will predominantly exist as the monomer form which yields green fluorescence signal. On the other hand, JC-1 aggregates together and yields red fluorescence in normal hyperpolarized mitochondria [28]. 

### 3.6. Western Blot Analysis

Cells were lysed in RIPA lysis buffer with protease and phosphatase inhibitors added to extract the total whole cell protein extract. The concentration of the total protein extract was determined by Bio-Rad DCTM protein assay kit (Bio-Rad, Philadelphia, PA, USA). Then 30 μg of total protein lysate were loaded and electrophoresed onto a 10% SDS-PAGE gel and then the separated proteins were transferred to a Nitrocellulose (NC) membrane. Membranes were blocked with 5% milk without fat in TBST for 1 h at room temperature. Primary anti-bodies (1:500 dilutions) were incubated overnight at 4 °C. After washing the membrane by TBST for three times (5 mins/time), secondary fluorescent antibody (1:10000 dilutions) were added to membrane at room temperature for 1 hr. Actin was used as the loading control and for normalization. The signal intensity of the membranes was detected by an LI-COR Odessy scanner (Belfast, ME, USA).

### 3.7. Statistical Analysis

All of the data were expressed as mean ± SE of three individual experiments. Differences between groups were determined by one way analysis of variance (ANOVA), followed by Bonferroni’s test to compare all pairs of columns. For all the tests, the level of significance was set at *p* < 0.05.

## 4. Conclusions

Recently, EGFR TKI, like gefitinib, is emerging as an effective clinical therapeutic drug for NSCLC patients who show EGFR overexpression or activated mutation on their lung tumor. However, the clinical success of EGFR TKI is limited by the development of drug resistance within 12 months. New effective agents for gefitinib-resistant patients are urgently required. Celastrol, which is a cytotoxic agent, exhibits significant anti-cancer activity in various cancer models [37], but its anti-cancer effect on gefitinib-resistant NSCLC cells remains unknown. In this study, we demonstrated that celastrol is an effective and potent agent in gefitinib-resistant NSCLC H1650 and H1975 cell lines. Celastrol exerted cytotoxicity in these two gefitinib-resistant NSCLC cell lines by induction of caspase-dependent apoptosis and Hsp90 client protein degradation.

Several apoptotic characteristics were observed in celastrol-treated H1650 and H1975 cells, such as cellular shrinkage, the cleavage of PARP and caspases activation. It has been reported that apoptosis signaling can be initiated either at the cell surface through a death receptor-induced signaling pathway, or within the cell via the release of pro-apoptotic factors such as cytochrome c and Bcl-2 family proteins [38]. In our study, we explored the apoptosis signaling pathways induced by celastrol in H1650; therefore MMP and the levels of some apoptotic-associated proteins were examined. In Figure 3, we observed that MMP was gradually lost, the ratio of Bax/Bcl-2 increased and cytochrome c was released from mitochondria by the treatment of celastrol. All these results suggested that celastrol induced a mitochondria-initiated apoptosis. In the mitochondria-initiated pathway, caspase activation is triggered by the formation of the Apaf-1/cytochrome c complex, which recruits and activates pro-caspase-9. Activated caspase-9 cleaves and activates downstream caspases, such as caspase-3, and -7 [39]. These are consistent with the results as shown in Figure 4. Moreover, we found that caspase-8 which is the key regulator of extrinsic apoptosis pathway was also remarkably activated. Next, a caspases inhibition experiment showed that pan-caspase inhibitor can partially attenuate the cytotoxic effect of celastrol, which indicated that caspases activation was involved in the apoptotic process induced by celastrol. However, the partial inhibition results also suggested that besides the induction of caspases activation, there could be another signaling pathway involved in celastrol-mediated apoptosis.

In several studies, celastrol has been shown to have strong anti-cancer activity by inducing the degradation of Hsp90 client proteins [34]. The major function of Hsp90 is to maintain the stability and activity of multiple kinases, transcription factors and steroid receptors [40], many of which are dysregulated in human cancer, such as Raf-1, EGFR and AKT [41,42,43]. The results in Figure 5 showed that celastrol induced the degradation of EGFR and AKT, which is consistent with the previous reports [34]. Since the activating mutation of EGFR plays a central role in the dysregulation of gefitinib-resistant H1650 NSCLC cells [44], the degradation of EGFR will contribute remarkably to inhibit the proliferation of these EGFR survival dependent cancer cells. Although the treatment mechanism of celastrol has been reported in two NSCLC cell lines H1975 and A549 [45,46], our results further indicated a new treatment mechanism of celastrol in a selective type of EGFR survival dependent gefitinb-resistant lung cancer.

In conclusion, findings in the current studies indicate that celastrol possesses significant apoptotic induction effects in gefitinib-resistant cells, and caspases activation and Hsp90 client protein degradation play important roles in this apoptotic process. Celastrol may potentially be developed as a promising agent for treatment of gefitinib-resistant NSCLC patients in the future. 

## References

[1] Sung B., Park B., Yadav V.R., Aggarwal B.B. (2010). Celastrol, a triterpene, enhances TRAIL-induced apoptosis through the down-regulation of cell survival proteins and up-regulation of death receptors. J. Biol. Chem..

[2] Tao X., Cush J.J., Garret M., Lipsky P.E. (2001). A phase I study of ethyl acetate extract of the chinese antirheumatic herb Tripterygium wilfordii hook F in rheumatoid arthritis. J. Rheumatol..

[3] Li H., Zhang Y.Y., Huang X.Y., Sun Y.N., Jia Y.F., Li D. (2005). Beneficial effect of tripterine on systemic lupus erythematosus induced by active chromatin in BALB/c mice. Eur. J. Pharmacol..

[4] Kiaei M., Kipiani K., Petri S., Chen J., Calingasan N.Y., Beal M.F. (2005). Celastrol blocks neuronal cell death and extends life in transgenic mouse model of amyotrophic lateral sclerosis. Neurodegener Dis..

[5] Allison A.C., Cacabelos R., Lombardi V.R., Alvarez X.A., Vigo C. (2001). Celastrol, a potent antioxidant and anti-inflammatory drug, as a possible treatment for Alzheimer’s disease. Prog. Neuro-Psychopharmacol. Biol. Psychiatry.

[6] Kim D.Y., Park J.W., Jeoung D., Ro J.Y. (2009). Celastrol suppresses allergen-induced airway inflammation in a mouse allergic asthma model. Eur. J. Pharmacol..

[7] Pang X., Yi Z., Zhang J., Lu B., Sung B., Qu W., Aggarwal B.B., Liu M. (2010). Celastrol suppresses angiogenesis-mediated tumor growth through inhibition of AKT/mammalian target of rapamycin pathway. Cancer Res..

[8] Sethi G., Ahn K.S., Pandey M.K., Aggarwal B.B. (2007). Celastrol, a novel triterpene, potentiates TNF-induced apoptosis and suppresses invasion of tumor cells by inhibiting NF-kappaB-regulated gene products and TAK1-mediated NF-kappaB activation. Blood.

[9] Yang H., Chen D., Cui Q.C., Yuan X., Dou Q.P. (2006). Celastrol, a triterpene extracted from the Chinese “Thunder of God Vine,” is a potent proteasome inhibitor and suppresses human prostate cancer growth in nude mice. Cancer Res..

[10] Huang Y., Zhou Y., Fan Y., Zhou D. (2008). Celastrol inhibits the growth of human glioma xenografts in nude mice through suppressing VEGFR expression. Cancer Lett..

[11] Jemal A., Siegel R., Xu J., Ward E. (2010). Cancer statistics, 2010. CA-Cancer J. Clin..

[12] Chang A. (2011). Chemotherapy, chemoresistance and the changing treatment landscape for NSCLC. Lung Cancer.

[13] Tam I.Y., Leung E.L., Tin V.P., Chua D.T., Sihoe A.D., Cheng L.C., Chung L.P., Wong M.P. (2009). Double EGFR mutants containing rare EGFR mutant types show reduced *in vitro* response to gefitinib compared with common activating missense mutations. Mol. Cancer Ther..

[14] Leung E.L., Tam I.Y., Tin V.P., Chua D.T., Sihoe A.D., Cheng L.C., Ho J.C., Chung L.P., Wong M.P. (2009). SRC promotes survival and invasion of lung cancers with epidermal growth factor receptor abnormalities and is a potential candidate for molecular-targeted therapy. Mol. Cancer Res..

[15] Wong D.W., Leung E.L., So K.K., Tam I.Y., Sihoe A.D., Cheng L.C., Ho K.K., Au J.S., Chung L.P., Pik Wong M. (2009). The EML4-ALK fusion gene is involved in various histologic types of lung cancers from nonsmokers with wild-type EGFR and KRAS. Cancer.

[16] Jun H.J., Johnson H., Bronson R.T., de Feraudy S., White F., Charest A. (2012). The oncogenic lung cancer fusion kinase CD74-ROS activates a novel invasiveness pathway through E-Syt1 phosphorylation. Cancer Res..

[17] Hirsch F.R., Varella-Garcia M., Bunn P.A., di Maria M.V., Veve R., Bremmes R.M., Baron A.E., Zeng C., Franklin W.A. (2003). Epidermal growth factor receptor in non-small-cell lung carcinomas: Correlation between gene copy number and protein expression and impact on prognosis. J. Clin. Oncol..

[18] Selvaggi G., Novello S., Torri V., Leonardo E., de Giuli P., Borasio P., Mossetti C., Ardissone F., Lausi P., Scagliotti G.V. (2004). Epidermal growth factor receptor overexpression correlates with a poor prognosis in completely resected non-small-cell lung cancer. Ann. Oncol..

[19] Ono M., Kuwano M. (2006). Molecular mechanisms of epidermal growth factor receptor (EGFR) activation and response to gefitinib and other EGFR-targeting drugs. Clin. Cancer Res..

[20] Lee J.Y., Lee Y.M., Chang G.C., Yu S.L., Hsieh W.Y., Chen J.J., Chen H.W., Yang P.C. (2011). Curcumin induces EGFR degradation in lung adenocarcinoma and modulates p38 activation in intestine: The versatile adjuvant for gefitinib therapy. PLoS One.

[21] Sequist L.V., Waltman B.A., Dias-Santagata D., Digumarthy S., Turke A.B., Fidias P., Bergethon K., Shaw A.T., Gettinger S., Cosper A.K. (2011). Genotypic and histological evolution of lung cancers acquiring resistance to EGFR inhibitors. Sci. Transl. Med..

[22] Sudo M., Chin T.M., Mori S., Doan N.B., Said J.W., Akashi M., Koeffler H.P. (2013). Inhibiting proliferation of gefitinib-resistant, non-small cell lung cancer. Cancer Chemother. Pharmacol..

[23] Choi Y.J., Rho J.K., Jeon B.S., Choi S.J., Park S.C., Lee S.S., Kim H.R., Kim C.H., Lee J.C. (2010). Combined inhibition of IGFR enhances the effects of gefitinib in H1650: A lung cancer cell line with EGFR mutation and primary resistance to EGFR-TK inhibitors. Cancer Chemother. Pharmacol..

[24] Bivona T.G., Hieronymus H., Parker J., Chang K., Taron M., Rosell R., Moonsamy P., Dahlman K., Miller V.A., Costa C. (2011). FAS and NF-kappaB signalling modulate dependence of lung cancers on mutant EGFR. Nature.

[25] Kim H.P., Han S.W., Kim S.H., Im S.A., Oh D.Y., Bang Y.J., Kim T.Y. (2008). Combined lapatinib and cetuximab enhance cytotoxicity against gefitinib-resistant lung cancer cells. Mol. Cancer Ther..

[26] Koivunen J.P., Mermel C., Zejnullahu K., Murphy C., Lifshits E., Holmes A.J., Choi H.G., Kim J., Chiang D., Thomas R. (2008). EML4-ALK fusion gene and efficacy of an ALK kinase inhibitor in lung cancer. Clin. Cancer Res..

[27] Zou J., Chen Q., Jin X., Tang S., Chen K., Zhang T., Xiao X. (2011). Olaquindox induces apoptosis through the mitochondrial pathway in HepG2 cells. Toxicology.

[28] Liu T., Hannafon B., Gill L., Kelly W., Benbrook D. (2007). Flex-Hets differentially induce apoptosis in cancer over normal cells by directly targeting mitochondria. Mol. Cancer Ther..

[29] Gong K., Li W. (2011). Shikonin, a Chinese plant-derived naphthoquinone, induces apoptosis in hepatocellular carcinoma cells through reactive oxygen species: A potential new treatment for hepatocellular carcinoma. Free Radic. Biol. Med..

[30] Lakhani S.A., Masud A., Kuida K., Porter G.A., Booth C.J., Mehal W.Z., Inayat I., Flavell R.A. (2006). Caspases 3 and 7: Key mediators of mitochondrial events of apoptosis. Science.

[31] Boatright K.M., Salvesen G.S. (2003). Mechanisms of caspase activation. Curr. Opin. Cell. Biol..

[32] Maddika S., Booy E.P., Johar D., Gibson S.B., Ghavami S., Los M. (2005). Cancer-specific toxicity of apoptin is independent of death receptors but involves the loss of mitochondrial membrane potential and the release of mitochondrial cell-death mediators by a Nur77-dependent pathway. J. Cell Sci..

[33] Eeva J., Nuutinen U., Ropponen A., Matto M., Eray M., Pellinen R., Wahlfors J., Pelkonen J. (2009). The involvement of mitochondria and the caspase-9 activation pathway in rituximab-induced apoptosis in FL cells. Apoptosis.

[34] Chen G., Zhang X., Zhao M., Wang Y., Cheng X., Wang D., Xu Y., Du Z., Yu X. (2011). Celastrol targets mitochondrial respiratory chain complex I to induce reactive oxygen species-dependent cytotoxicity in tumor cells. BMC Cancer.

[35] Li Y., Zhang T., Schwartz S.J., Sun D. (2009). New developments in Hsp90 inhibitors as anti-cancer therapeutics: Mechanisms, clinical perspective and more potential. Drug Resist. Updat..

[36] Nomoto K., Tsuta K., Takano T., Fukui T., Yokozawa K., Sakamoto H., Yoshida T., Maeshima A.M., Shibata T., Furuta K. (2006). Detection of EGFR mutations in archived cytologic specimens of non-small cell lung cancer using high-resolution melting analysis. Am. J. Clin. Pathol..

[37] He D., Xu Q., Yan M., Zhang P., Zhou X., Zhang Z., Duan W., Zhong L., Ye D., Chen W. (2009). The NF-kappa B inhibitor, celastrol, could enhance the anti-cancer effect of gambogic acid on oral squamous cell carcinoma. BMC Cancer.

[38] Lee M.S., Chao J., Yen J.C., Lin L.W., Tsai F.S., Hsieh M.T., Peng W.H., Cheng H.Y. (2012). Schizandrin protects primary rat cortical cell cultures from glutamate-induced apoptosis by inhibiting activation of the MAPK family and the mitochondria dependent pathway. Molecules.

[39] Mao X., Yu C.R., Li W.H., Li W.X. (2008). Induction of apoptosis by shikonin through a ROS/JNK-mediated process in Bcr/Abl-positive chronic myelogenous leukemia (CML) cells. Cell Res..

[40] Moser C., Lang S.A., Stoeltzing O. (2009). Heat-shock protein 90 (Hsp90) as a molecular target for therapy of gastrointestinal cancer. Anticancer Res..

[41] Basso A.D., Solit D.B., Chiosis G., Giri B., Tsichlis P., Rosen N. (2002). Akt forms an intracellular complex with heat shock protein 90 (Hsp90) and Cdc37 and is destabilized by inhibitors of Hsp90 function. J. Biol. Chem..

[42] Schulte T.W., Blagosklonny M.V., Ingui C., Neckers L. (1995). Disruption of the Raf-1-Hsp90 molecular complex results in destabilization of Raf-1 and loss of Raf-1-Ras association. J. Biol. Chem..

[43] Sawai A., Chandarlapaty S., Greulich H., Gonen M., Ye Q., Arteaga C.L., Sellers W., Rosen N., Solit D.B. (2008). Inhibition of Hsp90 down-regulates mutant epidermal growth factor receptor (EGFR) expression and sensitizes EGFR mutant tumors to paclitaxel. Cancer Res..

[44] Guo A., Villen J., Kornhauser J., Lee K.A., Stokes M.P., Rikova K., Possemato A., Nardone J., Innocenti G., Wetzel R. (2008). Signaling networks assembled by oncogenic EGFR and c-Met. Proc. Natl. Acad. Sci. USA.

[45] Liu Z., Ma L., Wen Z.S., Hu Z., Wu F.Q., Li W., Liu J., Zhou G.B. (2013). Cancerous inhibitor of PP2A is targeted by natural compound celastrol for degradation in non-small-cell lung cancer. Carcinogenesis.

[46] Mou H., Zheng Y., Zhao P., Bao H., Fang W., Xu N. (2011). Celastrol induces apoptosis in non-small-cell lung cancer A549 cells through activation of mitochondria- and Fas/FasL-mediated pathways. Toxicol. In Vitro.

